# Dynamic Spectral Modulation on Meta‐Waveguides Utilizing Liquid Crystal

**DOI:** 10.1002/advs.202304116

**Published:** 2023-10-23

**Authors:** Chengkun Dong, Ziwei Zhou, Xiaowen Gu, Yichen Zhang, Guodong Tong, Zhihai Wu, Hao Zhang, Wenqi Wang, Jun Xia, Jun Wu, Tangsheng Chen, Jinping Guo, Fan Wang, Fengfan Tang

**Affiliations:** ^1^ Joint International Research Laboratory of Information Display and Visualization School of Electronic Science and Engineering Southeast University Nanjing 210096 China; ^2^ National Key Laboratory of Solid‐State Microwave Devices and Circuits Nanjing Electronic Devices Institute Nanjing 210096 China; ^3^ Accelink Technology Co. Ltd. Wuhan 430010 China

**Keywords:** dynamic spectral modulation, forward scattering, integrated photonics, meta‐waveguides

## Abstract

The integration of metasurfaces and optical waveguides is gradually attracting the attention of researchers because it allows for more efficient manipulation and guidance of light. However, most of the existing studies focus on passive devices, which lack dynamic modulation. This work utilizes the meta‐waveguides with liquid crystal(LC) to modulate the on‐chip spectrum, which is the first experimentally verified, to the authors' knowledge. By applying a voltage, the refractive index of the liquid crystal surrounding the meta‐waveguides can be tuned, resulting in a blue shift of the spectrum. The simulation shows that the 18.4 dB switching ratio can be achieved at 1550 nm. The meta‐waveguides are prepared using electron beam lithography (EBL), and the improved transmittance of the spectrum in the short wavelength is experimentally verified, which is consistent with the simulation trend. At 1551.64 nm wavelength, the device achieves a switching ratio of ≈16 dB with an active area of 8 µm × 0.4 µm. Based on this device, an optoelectronic computing architecture for the Hadamard matrix product and a novel wavelength selection switch are proposed. This work offers a promising avenue for on‐chip dynamic modulation in integrated photonics, which has the advantage of a compact active area, fast response time, and low energy consumption compared to conventional thermal‐light modulation.

## Graphical abstract

This study reports for the first time the modulation of the on‐chip spectrum utilizing the meta‐waveguides with liquid crystal. Compared with traditional thermal‐optic modulation, it has the advantage of a compact active area, fast response time, and low energy consumption. Further, an optoelectronic computing architecture and a novel wavelength selection switch are proposed based on this device.

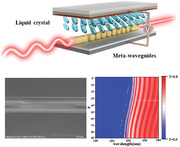

## 1 Introduction

With the progressive advancement of integrated photonics, a plethora of application scenarios have emerged,^[^ ^1^, ^2^, ^3^ ^]^ with optical communication,^[^ ^4^, ^5^, ^6^ ^]^ optical computing,^[^ ^7^, ^8^, ^9^ ^]^ light detection and ranging (LIDAR),^[^ ^10^, ^11^, ^12^ ^]^ and biosensing^[^ ^13^, ^14^, ^15^ ^]^ being the most notable and representative domains. Optical metasurfaces^[^ ^16^, ^17^, ^18^ ^]^ are ultra‐thin 2D planes composed of subwavelength metal or dielectric units that can flexibly modulate the amplitude, phase, and polarization of light at subwavelength scales, which are widely used in free‐space optics. Moreover, their integration with on‐chip optical waveguides presents novel opportunities and challenges for integrated photonics.^[^ ^19^, ^20^, ^21^ ^]^

Ding et al.^[^ ^22^ ^]^ experimentally verified the low‐loss slow light transmission of meta‐waveguides in the optical communication band, with low transmittance in the short‐wave band and high transmission efficiency in the long‐wave band. The recently published paper^[^ ^23^ ^]^ proposed that the transmittance of the meta‐waveguide will be enhanced when the subwavelength structures are designed so that the electric and magnetic dipolar Mie‐type resonances spectrally overlap over the full resonances. Based on this, two passive structures are proposed: bending waveguides and y‐branch waveguides.

Due to the limited functionality of passive devices, there have also been attempts on the active tunning of the metasurfaces.^[^ ^24^, ^25^, ^26^ ^]^ Han et al.^[^ ^27^ ^]^ utilized electrically tunable graphene metasurfaces to achieve complex amplitude modulation in the 5 µm band. Kim et al.^[^ ^28^ ^]^ confirmed that the electronically tunable perfect absorption in graphene can be achieved by graphene plasmonic nanostructures, with experimental results demonstrating a tunable wavelength range of 5–8 µm and a switch ratio of ≈96%. Phase‐change material (PCM) has been employed to realize programmable metasurfaces by applying an external stimulus, such as heat, optical pulses, or electrical pulses. Tripathi et al.^[^ ^29^ ^]^ and Ruiz De Galarreta et al.^[^ ^30^ ^]^ employed optical and electrical pulses, respectively, to excite phase‐change materials, thereby enabling dynamic modulation of metasurfaces. The method of thermal‐light tuning has been reported in Ref. [31], realizing the function of beam steering. The tunable metasurfaces with external stimuli mentioned in this part are summarized in **Table** **1**.

**Table 1:** The comparison of tunable metasurface with external stimuli.

Materials	External stimuli	Modulation speed	Wavelength	Switch ratio/angle of deflection	Compatibility with existing processes
graphene	Electrical^[^ ^27^ ^]^	NA	8 µm	≈100%	Average
	Electrical^[^ ^28^ ^]^	≈1 MHz	5‐8 µm	15.08dB	Average
Phase change materials	Optical^[^ ^29^ ^]^	NA	1.2‐1.6 µm	≈20dB	Poor
	thermal^[^ ^31^ ^]^	NA	2‐5 µm	14°	Average
	ELECTRICAL^[^ ^30^ ^]^	80 MHz	1.2–1.6 µm	5.22dB	Poor
Liquid crystal	Electrical (this work)	≈kHz	≈1.55 µm	≈16dB	High

Dynamic modulation of the phase of transmitted light was achieved using TiO_2_ metasurface wrapped with the liquid crystal(LC) in ref ^[^ ^32^ ^]^. The refractive index of the liquid crystal surrounding the metasurface is changed by applying a voltage, which causes a change in the Mie resonance properties of the metasurface. This feature enables the device to achieve 2π phase modulation with an ultra‐thin thickness of 665 nm, significantly improving the integration of the device. Park et al.^[^ ^33^ ^]^ dynamically modulated the optical response of the metasurface by changing the carrier concentration inside the material by applying a voltage.

However, the aforementioned dynamic modulations of metasurface are for spatial light fields, and on‐chip dynamic modulations are less reported. Ding et al.^[^ ^22^ ^]^ used picosecond lasers to pump meta‐atoms to achieve on‐chip high‐speed intensity modulation, and the device response time was less than 50 ps. The disadvantage of such an approach is that it relies on a large number of bulk optics, which is not conducive to the integration of the system.

Zhou et al.^[^ ^34^ ^]^ proposed the on‐chip tunable mode converter using liquid crystal, which has a compact footprint and low insertion loss. Advantages of integrating meta‐waveguides with liquid crystal over other dynamically modulated schemes include lower cost, compatibility with mature LCOS(Liquid Crystal On Silicon) processes, and facilitation of large‐scale integration. However, no real liquid crystal device was prepared for the experimental verification, and the function of the mode converter was only verified in two different media (water and air).

In this paper, we have proposed and experimentally verified the dynamic spectral modulation based on meta‐waveguides using liquid crystal. Simulations and experiments show that liquid crystal enhances the forward scattering of meta‐atom, and the addition of voltage makes the spectrum blueshift. The switching ratio of ≈16 dB is achieved at 1551.64 nm, and the area of the device is 8 µm×0.4 µm. The device exhibits a response time in the millisecond range. Since the devices have opposite modulation amounts at 1546 and 1550 nm, we propose a new optoelectronic architecture for Hadamard matrix products based on the proposed meta‐waveguides. In addition, the spectral shift characteristics of the device can enable it to be used as a wavelength selection switch. The fabrication of meta‐waveguides can be done by CMOS‐compatible^[^ ^35^ ^]^ photolithography, while liquid crystal can be pixelated and precisely regulated by LCOS ,^[^ ^36^ ^]^ which is of great importance for further integration of such devices.

## 2 Results

### 2.1 Design and Simulation

**Figure 1:**
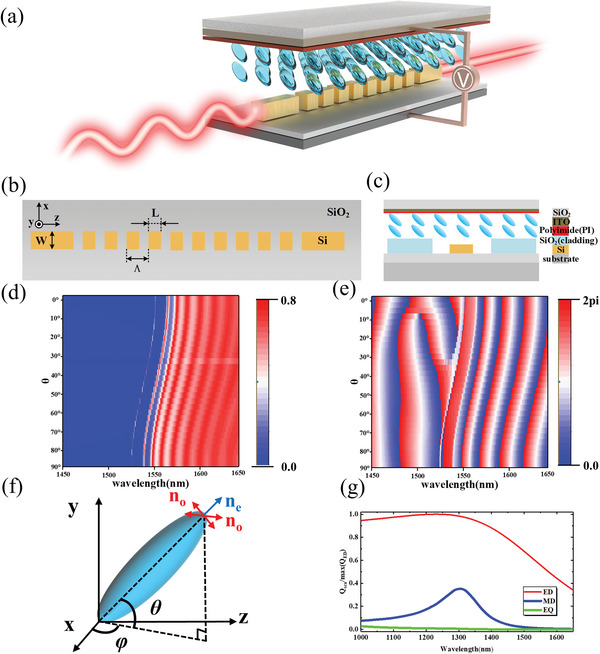
(a) Schematic of the tunable meta‐waveguides using liquid crystal. (b) Top view and (c) cross‐sectional view. (d) Transmission and (e) phase as a function of wavelength and LC orientation. Panel (f) shows the liquid crystal deflection angle *θ* and orientation angle *φ*. (g) Multipolar decomposition of single meta‐atom, which are normalized to the maximum of the ED contribution.

The proposed meta‐waveguide is composed of 40 silicon nanoparticles with a fixed height (H) of 220 nm, width (W) of 420 nm, and period (Λ) of 400 nm, as shown schematically in **Figure** **1**a. The length (L) follows a quadratic function distribution and ranges from 205 to 243 nm. The liquid crystal layer is between the meta‐waveguide and the ITO glass. The orientation film is only present on ITO glass, and the orientation of liquid crystal molecules is perpendicular to the waveguide direction. In this work, the orientation layer is polyimide (PI). Due to the anchoring effect of the meta‐waveguides^[^ ^37^ ^]^ on liquid crystal molecules, the arrangement of the lower liquid crystal molecules is also perpendicular to the waveguide direction. The top and cross‐sectional views of the device are shown in Figure 1b,c.

The electromagnetic field distribution of the structure is analyzed by FDTD. The mode in the waveguide is set as TE fundamental mode in the simulation, and the perfectly matched layer boundary condition is used to avoid reflection and absorb the light wave that crosses the boundary. The liquid crystal molecules point in the *x*‐direction when not charged, and in the *y*‐direction under the applied electric field. The transmittance and phase as a function of wavelength and LC orientation are shown in Figure 1d,e. The light in the meta‐waveguides is along the *z*‐axis while the liquid crystal rotates and always keeps the long axis parallel to the xoy plane, and there is no change in the refractive index of the liquid crystal along the direction of light propagation after applying an electric field. Figure 1g illustrates the results of multipole decomposition for the Mie resonant modes inside a single meta‐atom in the meta‐waveguides, including the electric dipole (ED), magnetic dipole (MD), and electric quadrupole (EQ)^[^ ^38^, ^39^ ^]^ As can be seen from Figure 1g, the single meta‐atom in the meta‐waveguides operates at their fundamental ED resonance in the 1550 nm band.

The dielectric constant of the liquid crystal after rotation is determined by two angles (*θ* and *φ*, as shown in Figure 1f):

(1) $$\varepsilon_{\mathrm{LC}-\mathrm{RO}}=U^{*}\left(\theta ,\varphi \right)\varepsilon_{\mathrm{LC}}U\left(\theta ,\varphi \right)$$

(2) $$\varepsilon_{\mathrm{LC}}=\left(\begin{array}{ccc} n_{o}^{2} & 0 & 0\\ 0 & n_{o}^{2} & 0\\ 0 & 0 & n_{e}^{2} \end{array}\right)$$

(3) $$\begin{array}{ccc} U(\theta ,\varphi ) & = & U_{z}U_{y}\\ & = & \left(\begin{array}{ccc} \cos\varphi & -\sin\varphi & 0\\ \sin\varphi & \cos\varphi & 0\\ 0 & 0 & 1 \end{array}\right)\left(\begin{array}{ccc} \cos\left(\frac{\pi }{2}-\theta \right) & 0 & \sin\left(\frac{\pi }{2}-\theta \right)\\ 0 & 1 & 0\\ -\sin\left(\frac{\pi }{2}-\theta \right) & 0 & \cos\left(\frac{\pi }{2}-\theta \right) \end{array}\right)\\ & = & \left(\begin{array}{ccc} \cos\left(\frac{\pi }{2}-\theta \right)\cos\varphi & -\sin\varphi & \sin\left(\frac{\pi }{2}-\theta \right)\cos\varphi \\ \cos\left(\frac{\pi }{2}-\theta \right)\sin\varphi & \cos\varphi & \sin\left(\frac{\pi }{2}-\theta \right)\sin\varphi \\ -\sin\left(\frac{\pi }{2}-\theta \right) & 0 & \cos\left(\frac{\pi }{2}-\theta \right) \end{array}\right) \end{array}$$

In the formula, *n*_e_ is 1.746, n_o_ is 1.521, *φ* is the angle to the *z*‐axis (xoz plane), and *θ* is the angle to the *x*‐axis (xoy plane). *θ* represents the deflection angle of the liquid crystal after being powered on, and *φ* represents the orientation angle, which is taken as 90° in this paper. The transformation matrix *U_y_* represents a rotation around the *y*‐axis, while the transformation matrix *U_z_* represents a rotation around the *z*‐axis. The transformation matrix *U*(*θ, φ*) represents a simultaneous rotation around both the *y*‐axis and the *z*‐axis, which is related to *θ* and *φ*. ε*_LC_* represents the dielectric constant of the liquid crystal, and ε_*LC*∐*RO*_ represents the dielectric constant of the liquid crystal after rotation.

**Figure 2:**
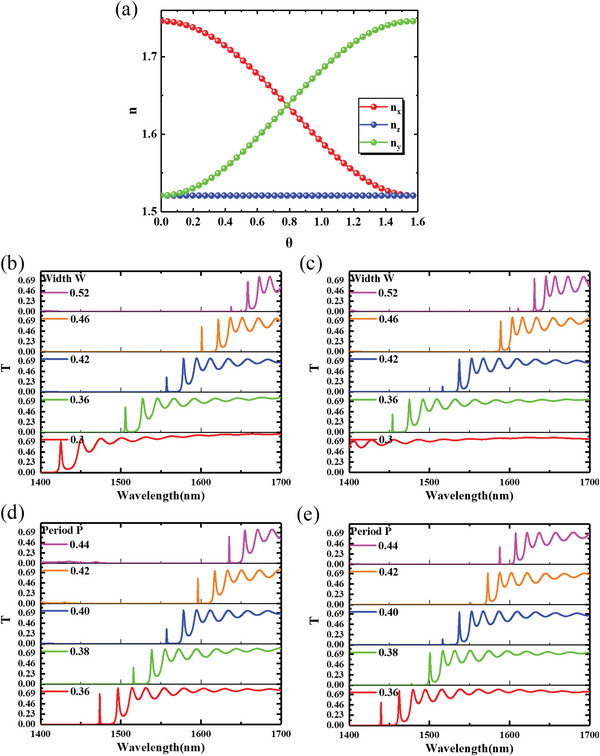
(a) n_x_, n_y_, and n_z_ as function of *θ*. Panels (b) and (c) show the transmission spectrum of meta‐waveguides at liquid crystal deflection angles of 0° and 90° with different widths. Panels (d) and (e) show the transmission spectrum of meta‐waveguides at liquid crystal deflection angles of 0° and 90° with different periods.

Based on the equation of the dielectric constant of the liquid crystal with respect to *θ* and *φ*, we derive the relationship between the refractive index components in different directions and the deflection angle of the liquid crystal, as shown in **Figure** **2**a. When a voltage is applied, the liquid crystal deflection angle *θ* changes from 0° to 90°, the *x*‐direction refractive index (n_x_) decreases from 1.746 to 1.521, the *z*‐direction refractive index (n_z_) increases from 1.521 to 1.746, and the *y*‐direction refractive index (n_y_) remains unchanged.

The transmittance of meta‐waveguides is basically 0 in the short wavelength range, while it is higher in the long wavelength range. As the angle between the direction vector of the liquid crystal and the *z*‐axis decreases, the overall spectrum shows a blue shift trend. At a wavelength of 1550 nm, when the liquid crystal director approaches parallel to the *z*‐axis, a transmission peak of light appears at that wavelength. When the liquid crystal director approaches parallel to the *x*‐axis, light is prohibited from passing through at that wavelength. The simulation shows that the switching ratio of the meta‐waveguides can reach 18.4 dB at 1550 nm.

Figure 2b–e exhibits the transmission spectra at different liquid crystal deflection angles for different widths (0.36, 0.38, 0.42, 0.46, and 0.52 µm) and periods (0.36, 0.38, 0.40, 0.42, and 0.44 µm) of meta‐atom. As shown in the figure, the spectrum undergoes a red shift as the width and period increase. When the width is 0.42 µm and the period is 0.4 µm, the transmittance of the meta‐waveguides at the wavelength near 1550 nm exhibits the most significant variation when the liquid crystal is deflected.

### 2.2 Device Fabrication and Characterization

**Figure 3:**
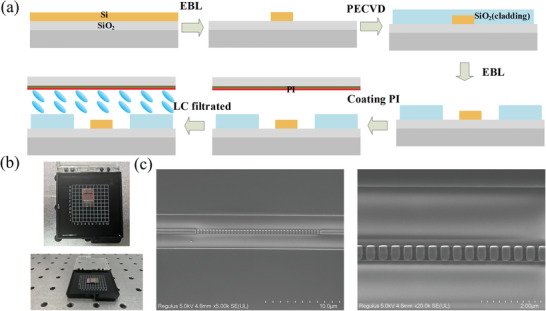
(a) Flow chart of device preparation. Panel (b) shows the top and side views of the device. Panel (c) shows the SEM images of the meta‐waveguide.

All the proposed devices were fabricated on the SOI substrate. The flow chart of the device preparation is shown in **Figure** **3**a. As a first step, the coupling gratings and meta‐waveguide were patterned by electron beam lithography(EBL) (Elionix els125‐g8) and etching. Second, a 2‐micron thick oxide silicon layer was deposited using PECVD. Using a combination of dry etching and wet etching, the oxide silicon above the nanoantenna was removed to enable the nanoantenna to be wrapped in liquid crystal.

Then fabrication of the meta‐waveguide with liquid crystal is fully compatible with the LCOS manufacturing process. Finally, the liquid crystal is filled between the substrate and ITO glass. The top view and side view of the final tested device are shown in Figure 3b. The morphology of the device was characterized by high‐resolution scanning electron microscopy as Figure 3c.

The device is powered on as follows: The electrode is grounded on a silicon substrate, and a high level is applied to the ITO. The amplitude of electrical pulses with a square waveform at 1 kHz frequency varies from 0 V to 10 V. Light is coupled from a single‐mode fiber into a waveguide, and then coupled again through another fiber to an intensity detector or spectrometer.

### 2.3 Experiment

**Figure 4:**
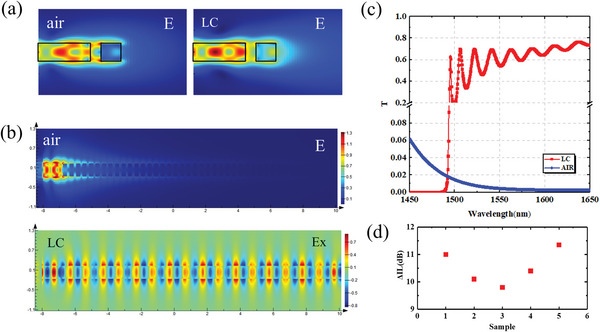
(a) The mode field of the single meta‐atom. (b) The mode field transmission of the meta‐waveguide simulated by FDTD in air and liquid crystal. (c) Device transmittance at 1450–1650 nm simulated by FDTD in air and liquid crystal. (d) Measured device insertion loss reduction after packaging liquid crystal.

Before preparing the LC cell, we measured the transmittance of the meta‐waveguides in the 1550 nm band and compared it with the transmittance of the meta‐waveguides wrapped in the liquid crystal. **Figure** **4**a shows the mode field of the single meta‐atom simulated by FDTD. When the medium around the meta‐atom is air, the forward scattering is significantly weaker than in liquid crystal. Figure 4b shows the mode field transmission diagrams in air and liquid crystal, respectively. The simulations show that the optical field cannot be transmitted in the meta‐waveguide, when the medium is air. Figure 4c shows the transmittance of the meta‐waveguide at 1450–1650 nm, which covers the S band, C band, L band, and a part of the U band.

Figure 4d shows the comparison of actual measured transmittance in the 1550 nm wavelength band. In order to facilitate liquid crystal packaging and coupling fiber testing,^[^ ^40^ ^]^ the waveguide length is about 1.5 cm. Due to the process, the insertion loss of different waveguides will vary. Figure 4d shows the comparison of actual measured transmittance in the 1550 nm wavelength band. Here we prepared five waveguides, and the insertion loss of the meta‐waveguides is reduced by more than 10 dB after liquid crystal wrapping, as shown in Figure 4d. This is due to the high refractive index of liquid crystal compared to air, which enhances the resonance effect of the metasurface^[^ ^41^ ^]^ and facilitates the forward scattering of the light field.^[^ ^42^ ^]^ It is also demonstrated that the liquid crystal is coupled to the metasurface, laying the foundation for modulating the spectrum by adding voltage to rotate the liquid crystal.

After preparing the LC cell, we performed a preliminary power‐up test on the device in free space (without fiber coupling) to ensure that the LC works. We place the device between two orthogonal linear polarizers and use a laser to irradiate the device. The reflected light is presented on a white screen. After adding a square wave signal with different voltages of 1 kHz, the light spots on the white screen have light and dark changes, ensuring that the LC can rotate after being powered on. The experimental setup and results are presented in Figure S2 (Supporting Information).

**Figure 5:**
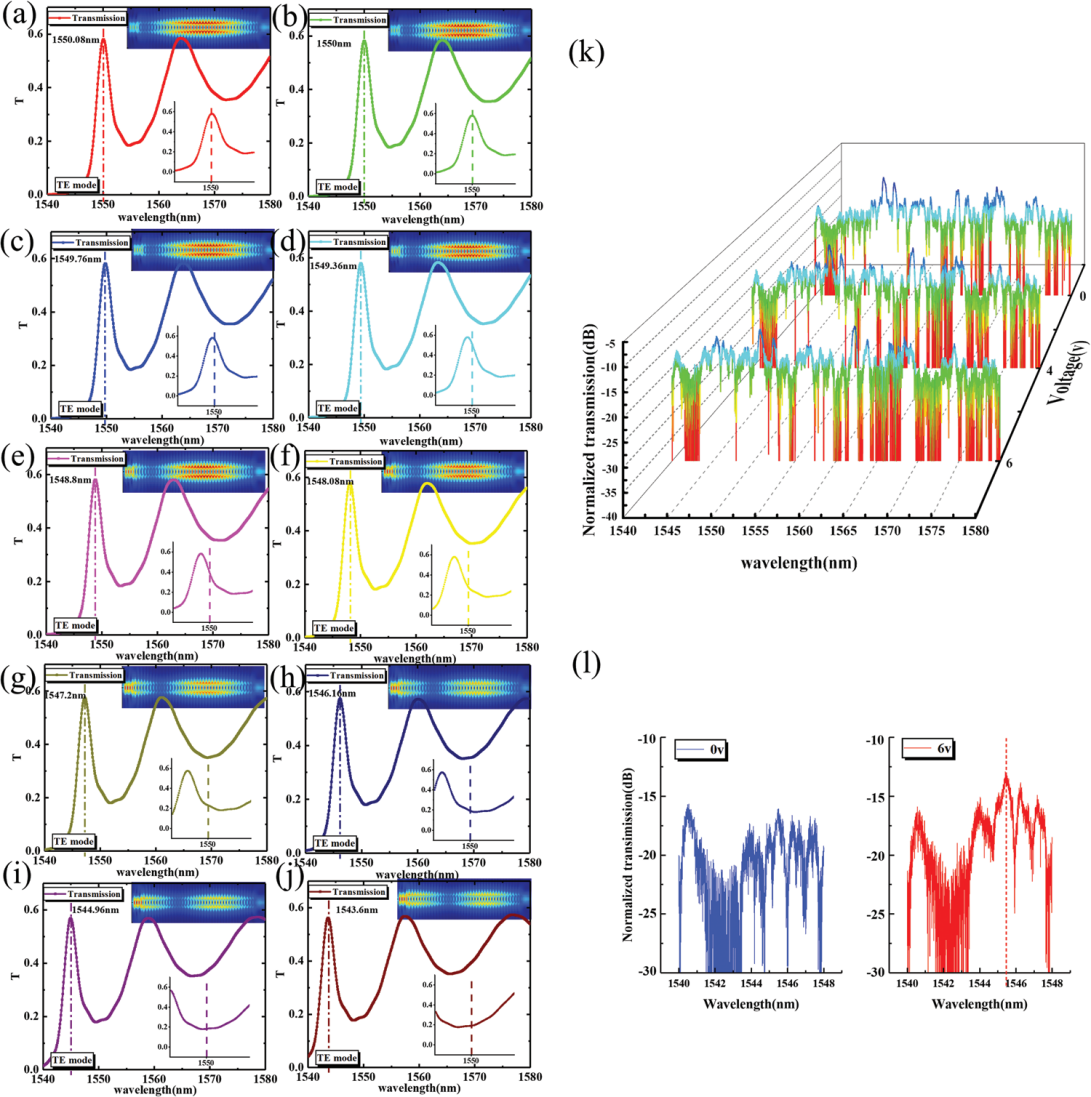
(a‐j) The spectrum variation of the meta‐waveguide with LC rotates. The inset shows the corresponding mode field diagram in 1550 nm. (k) The measured spectrum of the overall on‐chip system (including meta‐waveguide, coupling gratings, and tapers) at different voltages. (l) Spectrum of the device in the wavelength range from 1540 to 1548 nm at 0 and 6 V.

Due to process limitations, our actual meta‐waveguide width is 400 nm and length is 250 nm. **Figure** **5**a–j shows the simulated transmission spectrum of the meta‐waveguide with a decreasing angle between liquid crystal molecules and the *z*‐axis as the voltage increases. From the simulation results, it can be seen that the spectrum undergoes a blue shift with increasing voltage, and the resonance peak near 1550 nm (when not powered on) shifts toward the short wavelength. Figure 5k shows the measured spectrum of the device at voltages of 0, 4, and 6 V. It is worth noting that the transmission spectrum of the entire system includes coupling gratings, tapers, and meta‐waveguides. The transmission spectrum of the meta‐waveguide changes with the applied voltage, while the other parts are coated with silica, and the spectral response remains unchanged. The input light source is an ASE light source, and the spectral detection range is 1540–1580 nm. From Figure 5k, it can be seen that the transmittance of the short wavelength band gradually increases as the voltage increases, which is consistent with the simulation trend. The transmittance spectrum at different voltages from 1540 to 1548 nm is shown in Figure 5l. As can be seen from the figure, a transmission peak appears near 1545 nm after applying voltage. Meanwhile, the transmittance in the 1550 nm band decreases with the shift of the peak as the voltage increases.

**Figure 6:**
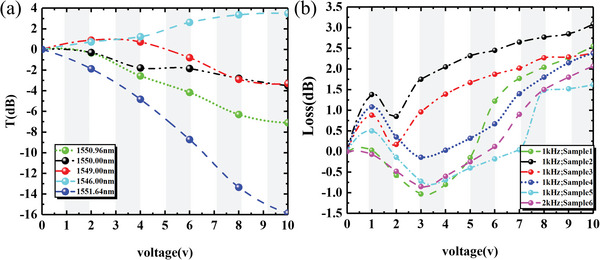
(a) The variation of meta‐waveguides transmittance at different bands at 0, 2, 4, 6, 8, and 10 V according to the test spectrum. (b) The transmittance of the meta‐waveguide at 1550 nm was repeatedly tested using a monochromatic laser.

**Figure** **6**a shows the variation of meta‐waveguides transmittance at 1546.00, 1549.00, 1550.00, 1550.96, and 1551.64 nm. From the results, the proposed device achieved a switching ratio of more than 15 dB at 1551.64 nm, and the transmittance at 1550 and 1546 nm has the opposite trend with the applied voltage.

Further, we coupled a 1550 nm monochromatic laser into the device using optical fiber, and used an optical power meter to measure the output light intensity of the device under different voltages. The experimental results of multiple measurements are shown in Figure 6b.

It can be seen from the spectrum variation with the voltage diagram that the device will have completely opposite modulation effects in two specific bands. For example, when the voltage is from 0 to 10 V, the transmittance of the device will decrease by ≈3.5 dB in the band 1550 nm, while the transmittance of the device will increase by ≈3.5 dB in the band 1546 nm. The modulation effect is similar to that of optical inverters, which makes the device useful in optical computing and optical information processing systems. Here, we use the device for compressed sensing imaging. One of the observation matrix types for compressed sensory imaging is the Hadamard matrix, and any two columns or rows of the Hadamard matrix^[^ ^43^, ^44^ ^]^ satisfy the mutual orthogonality relationship, and the matrix is composed entirely of +1 or −1 elements. Therefore, this computational process can be done by this device.

**Figure 7:**
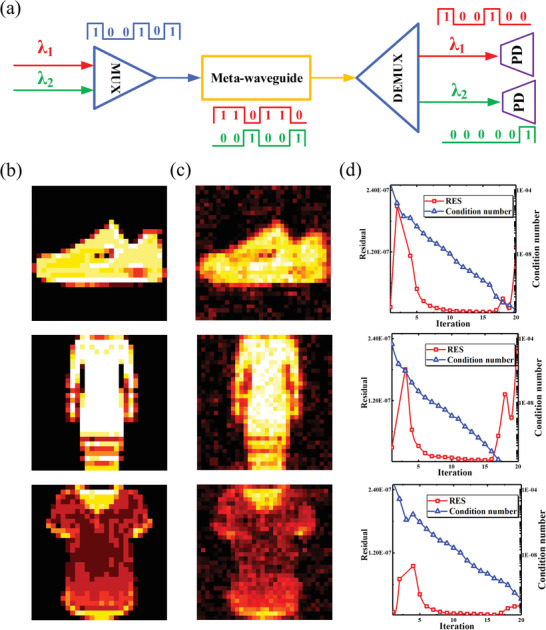
(a) Flow chart of Hadamard product implementation using the proposed meta‐waveguide with the liquid crystal. Blue line: input signal; Red and green line: modulation amount and output results for different bands. (b) Ground truth. (c) Reconstructed images. The RES and Condition number in the iterative process are shown in panel (d).

We perform a compression sensing imaging^[^ ^45^, ^46^ ^]^ simulation based on the actual measured physical parameters of the device. The process is shown in **Figure** **7**a. The pixel grayscale data of a 2D image is flattened into a one‐dimensional vector and loaded simultaneously on two wavelengths (1546 and 1550 nm). The beam is combined after passing through a wavelength division multiplexer^[^ ^47^ ^]^ and modulated by the device. The device performs binary intensity modulation on the input light field, with a modulation voltage of 0 or 10 V. The modulation value of the device is determined based on the value of the target Hadamard matrix. Due to the opposite amplitude modulation effect of the device on two wavelengths, that is −3.5 dB at 1550 nm and +3.5 dB at 1546 nm, the modulated signals at the two wavelengths will have different results. After being modulated by the proposed device, the signal is separated using demux and then converted into two electrical signals using photodetectors. The subtracted value of the two electrical signals is the product of the input matrix and the Hadamard matrix (The process is presented in Figure S4b, Supporting Information).

In order to be closer to the real situation, we add Gaussian noise in the process of multiplying with the Hadamard matrix. Figure 7b shows the ground truth, and Figure 7c shows the results of compressed perceptual imaging. Figure 7d shows the Residual and Condition numbers in the iterative process, which indicates the result converges.

The change of the refractive index of the liquid crystal, as mentioned earlier, can cause a shift in the spectrum of the meta‐waveguides. Based on simulation results, the device can achieve the function of dynamic wavelength selection,^[^ ^48^, ^49^ ^]^ which is widely used in wavelength‐division multiplexing (WDM) systems. This is because in the WDM system, the optical signal needs to be separated first, so that the optical signal of different wavelengths can be sent to different receivers.

**Figure 8:**
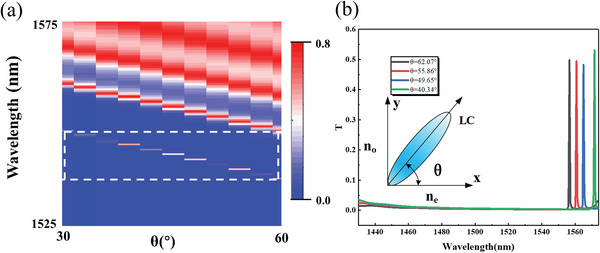
(a) The transmission peak near 1560 nm (mark it with white dashed lines) moves with the rotation of liquid crystal.(b) The spectrum of meta‐waveguides from 1430 to 1570 nm at the liquid crystal deflection angle *θ* of 40.34°, 49.65°, 55.86°, and 62.07°.

**Figure** **8**a shows the transmission spectrum of the device in the wavelength range of 1525–1575 nm when the liquid crystal deflection angle is between 30° and 60°. As shown in Figure 8a, the transmission spectrum of the device has only a single peak in the wavelength near 1560 nm. Within this range, only the transmittance at the peak is relatively high, and the transmittance at other wavelengths is close to 0. Therefore, the wavelength at the peak can be selected.

Furthermore, the spectrum shifts as the voltage is applied, resulting in a corresponding shift of the transmission peak. Figure 8b shows the position of the corresponding peak value under different liquid crystal deflection angles. At the liquid crystal deflection angles of 40.34°, 49.65°, 55.86°, and 62.07°, wavelengths of 1556.6, 1560.8, 1565.0, and 1571.6 nm are selected. From the figure, it can be seen that when the liquid crystal deflection angle rotates, the target wavelength will be dynamically selected after modulation by the device.

## 3 Conclusion

In conclusion, we have proposed and experimentally verified the dynamic spectral modulation based on meta‐waveguides using liquid crystal. We simulated the optical properties of meta‐waveguides, and then prepared them using microfabrication processes such as EBL. Both simulation and experiment have verified the blue shift of the spectrum after applying voltage. The dynamic modulation of the on‐chip spectrum is realized with a compact size, and a switching ratio of ≈16 dB was experimentally realized. The device has a fast response time and low power consumption, which is expected to be used in the field of optical computing or optical communication.

## Conflict of Interest

The authors declare no conflict of interest.

## Supporting information

Supporting Information

Supplemental Video 1

Supplemental Video 2

Supplemental Video 3

## Data Availability

The data that support the findings of this study are available from the corresponding author upon reasonable request.
