# Supplementary material for: Dynamic Spectral Modulation on Meta‐Waveguides Utilizing Liquid Crystal
Source: Adv Sci (Weinh). 2023 Oct 23;10(34):2304116. doi: 10.1002/advs.202304116 (PMC10700212; doi:10.1002/advs.202304116)
Supplement: Supplementary file 1 — Supporting Information [file ADVS-10-2304116-s002.pdf]

ADVANCED SCIENCE
Open Access

Supporting Information

for *Adv. Sci.*, DOI 10.1002/advs.202304116

Dynamic Spectral Modulation on Meta-Waveguides Utilizing Liquid Crystal

Chengkun Dong, Ziwei Zhou, Xiaowen Gu, Yichen Zhang, Guodong Tong, Zhihai Wu, Hao Zhang, Wenqi Wang, Jun Xia, Jun Wu, Tangsheng Chen, Jinping Guo, Fan Wang and Fengfan Tang*

Supplementary Material for

Dynamic spectral modulation on meta-waveguides utilizing liquid crystal

Chengkun Dong[1], Ziwei Zhou[1], Xiaowen Gu[1,2,3], Yichen Zhang[1], Guodong Tong[1], Zhihai Wu[1], Hao Zhang[1], Wenqi Wang[1], Jun Xia[1]*, Jun Wu[1], Tangsheng Chen[1,2,3], Jinping Guo[4], Fan Wang[4], and Fengfan Tang[4].

1. *Joint International Research Laboratory of Information Display and Visualization, School of Electronic Science and Engineering, Southeast University, Nanjing 210096, China*
2. *National Key Laboratory of Solid-State Microwave Devices and Circuits, Nanjing 210000, China*
3. *Nanjing Electronic Devices Institute, Nanjing 210096, China*
4. *Accelink Technology Co. Ltd., Wuhan 430010, China*

1. Here, we use a coupling grating to couple the light from the single-mode fiber into the waveguide and use the coupling grating at the other end to couple the light modulated by the meta-waveguide into the photodetector or spectrometer. Below is the SEM plot and filtered spectrum of the coupled grating.

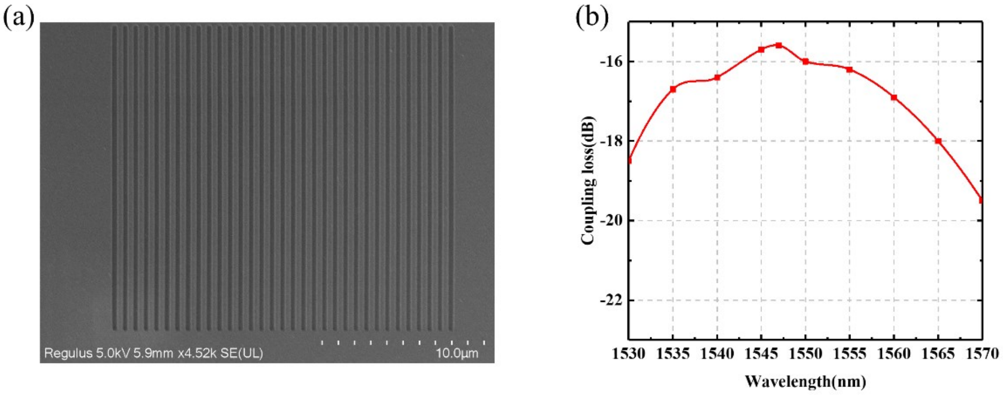

Figure s1. a) shows the SEM diagram of the coupled grating. b) shows the filtered spectrum of the coupled grating

2. The packaging process of liquid crystal box mainly includes cleaning glass, spin coating PI, orientation, curing, sealing, and filling liquid crystal. First, the ITO glass needs to be cleaned to prevent dirt from affecting the film quality. The PI used in this experiment is PIA-CE01-36X, which should be stored at -20°C. After completing the spin coating of pi, orientation, and curing are performed. This step evaporates the PI solvent, delaminates, and forms the film surface, and involves baking at 80°C for 140 seconds. The orientation of the pi was then performed using the optical alignment method, using a SunSpot2 UV light source with an Inventech optical alignment machine and a 365 nm filter to obtain the desired wavelength of UV light. The polarization was adjusted in the same direction as the orientation, and the power and duration were adjusted so that the exposure was 2 J/cm. The film is then baked at 110°C

for 20 minutes to enhance alignment. Finally, the PI film is cured by baking at 230°C for 20 minutes for the cyclization reaction. After completing the above steps, the upper substrate is prepared. Since the meta-waveguides were prepared on the lower substrate, PI was not spin-coated on the lower substrate, and then the upper and lower substrates were fixed by frame-sealing adhesive. The 3um spacers were mixed into the UV curing adhesive and stirred well to fix the thickness of the box. Then the E7 liquid crystal was dripped into the box through a capillary tube on a hot table at 40C. Finally, seal the remaining two sides in the same way to complete the production of the liquid crystal box.

3. Fig s2 shows an optical test of the dynamic performance of the device achieved by applying voltage. A laser with a wavelength of 532 nm passes through the P1 polarizer and then produces linear polarized light to the device. Since the device's substrate is silicon, it will reflect most of the incident light. Since P1 and P2 are completely orthogonal, only the light modulated by the liquid crystal region has energy left after the P2 polarizer when the device is not charged. When the device is powered up, the originally lit area gradually becomes darker, which indicates that the liquid crystal is gradually rotating and the device is working, as shown in video s1. Line polarized light becomes elliptically polarized light when it is incident on the liquid crystal device. The elliptically polarized light reflected from the liquid crystal region is projected onto a white screen after passing through a line polarizer. When the device is powered on, the liquid crystal molecules rotate, and the birefringence effect in the direction of light propagation is weakened. The light modulated by the liquid crystal device remains linearly polarized. When a voltage is applied, the spot on the white screen gradually changes from bright to dark, as shown in Fig s3. This indicates that the refractive index of the device changes after being powered on.

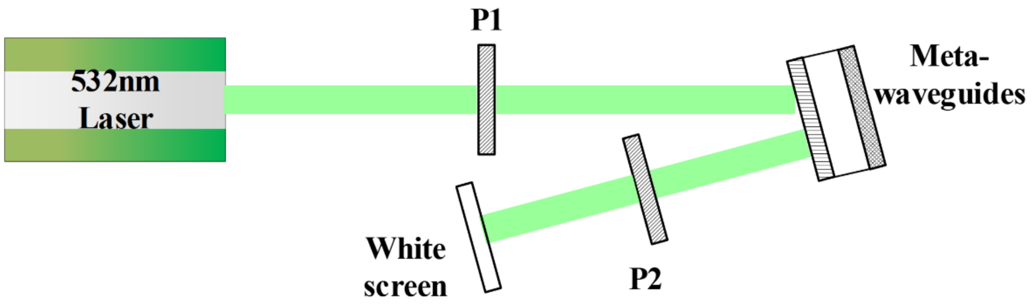

Figure s2. Optical characterization setup for the device. P1 and P2 are polarizers

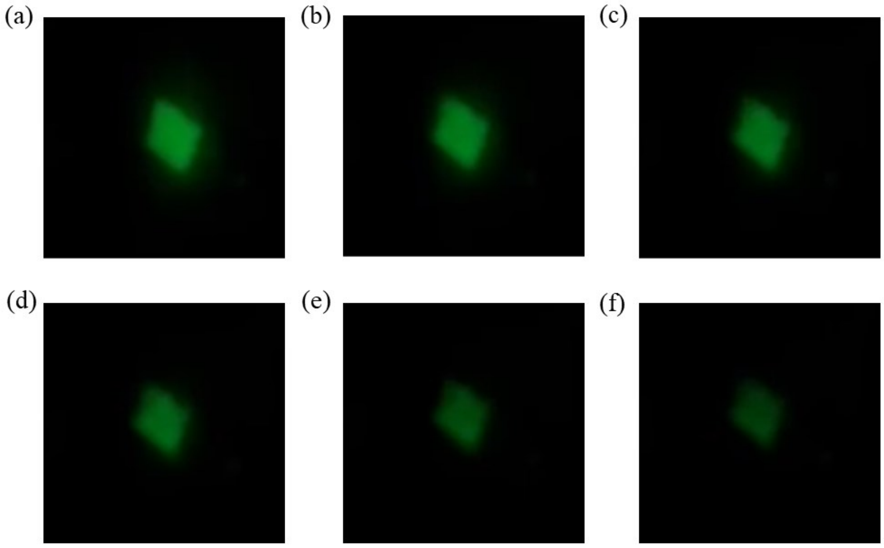

Figure s3. Spot brightness of linearly polarized light modulated by liquid crystal device at different voltages, a) 0v, b) 2v, c) 4v, d) 6v, e) 8v, and f) 10v.

3. Video s2 and s3 show the light field distribution of the meta-waveguide at different wavelengths for liquid crystal rotation angles of 90° and 0°. From the video s2 and s3, it can be seen that the spectrum undergoes a blue shift after being powered on

4. The theory of compressed sensing is that under the condition that the sampling rate is far less than Nyquist's, it uses random sampling to obtain discrete samples of the signal, and then perfectly reconstructs the signal through a nonlinear reconstruction algorithm. The process is shown in Figure s4 a) and b). The process of realizing the Hadamard matrix multiplication using the meta-waveguide is as follows: after completing the modulation of the two bands in the optical domain, the signal is converted to the electrical domain, and then the signal is subtracted bit by bit, thus realizing the matrix multiplication of the input signal and the Hadamard matrix.

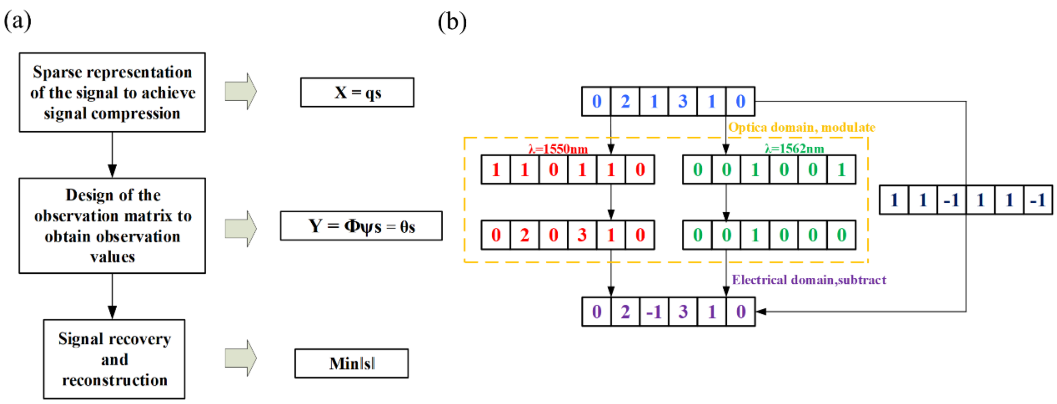

Figure s4. a) Flow chart of compressed sensing imaging. b) Flow chart of realizing product with Hadamard matrix.
